# Toll-Like Receptor 8 Agonist Strengthens the Protective Efficacy of ESAT-6 Immunization to *Mycobacterium tuberculosis* Infection

**DOI:** 10.3389/fimmu.2017.01972

**Published:** 2018-01-24

**Authors:** Jun Tang, Mengmeng Sun, Guiying Shi, Yanfeng Xu, Yunlin Han, Xiang Li, Wei Dong, Lingjun Zhan, Chuan Qin

**Affiliations:** ^1^Institute of Laboratory Animal Sciences, Chinese Academy of Medical Sciences (CAMS), Comparative Medicine Center, Peking Union Medical College (PUMC), Beijing, China; ^2^Tuberculosis Center, Chinese Academy of Medical Sciences (CAMS), Beijing, China; ^3^Key Laboratory of Human Disease Comparative Medicine, Ministry of Health, Beijing, China; ^4^Beijing Key Laboratory for Animal Models of Emerging and Reemerging Infectious Diseases, Beijing, China

**Keywords:** TLR8, tuberculosis, adjuvants, immunologic, memory T cell, type I IFN signaling

## Abstract

Accumulating evidence suggests important functions for human Toll-like receptor 8 *in vivo* in tuberculosis and autoimmune diseases. However, these studies are limited by the lack of specific agonists and by the fact that the homology of TLR8 in human and mice is not sufficient to rely on mouse models. In this study, we examined the role of human TLR8 in the disease progression of experimental *Mycobacterium tuberculosis (Mtb)* infection, as well as the benefits provided by a TLR8 agonist against *Mtb* challenge in a human *TLR8* transgenic mouse. We found that the expression of human TLR8 in C57BL/6 mice permits higher bacilli load in tissues. A vaccine formulated with ESAT-6, aluminum hydroxide, and TLR8 agonist provided protection against *Mtb* challenge, with a high percentage of CD44^hi^CD62L^hi^ T_CM_. Using ovalbumin as a model antigen, we demonstrated that the activation of TLR8 enhanced the innate and adaptive immune response, and provided a sustained T_CM_ formation and Th1 type humoral response, which were mainly mediated by type I IFN signaling. Further research is required to optimize the vaccine formulation and seek optimal combinations of different TLR agonists, such as TLR4, for better adjuvanticity in this animal model.

## Introduction

As a continued threat to global health, TB epidemics have caused 1.4 million deaths per year. The spread of multidrug-resistant TB increases the difficulty for prevention and treatment. A new TB vaccine is potentially the most powerful tool to end TB, and seeking new adjuvants is one important direction toward its development.

Many toll-like receptor ligands have been synthesized and discovered, and many of them have been confirmed as promising adjuvant candidates. Agonists of TLR2 (1), TLR4 (2, 3) (GLA-SE, AS01), and TLR9 (IC31) (4) have all demonstrated the ability to increase the protective effect of the *Mtb* vaccine. Agonists of TLR7/8 have also been investigated as adjuvants and are considered to improve the effect of vaccine formulation. In most studies, owing to the similarity in function of the ligands, the adjuvant effect of activating TLR8 was always evaluated along with TLR7 by TLR7/8 ligands such as R848 (5). However, the difference between TLR7 and TLR8, as well as the interaction between these two receptors (6), render the *in vivo* effect of specifically activating TLR8 unknown. Furthermore, it is well known that the function of TLR8 in mice is different from the human analog (7). Although the similarity between TLR7 and TLR8 is quite high, more agonists with high specificity and potency are being discovered, such as VTX-2337 (Motolimod), which has entered into a phase Ib trial to treat recurrent or metastatic SCCHN patients with cetuximab (8). However, the homology of TLR8 in human and mouse is not high enough to rely on a mouse model. Therefore, several types of human *TLR8* transgenic or humanized mice were produced to study its function *in vivo*.

*TLR8* knock-in mice (9) or *TLR8* transgenic mice under the control of human *TLR8* genomic regulatory regions (7) tend to develop phenotypes correlated with autoimmune responses, such as systematic inflammations. The severity of the phenotypes closely correlates with the expression level of TLR8 (7). These mouse models help us observe how human TLR8 functions *in vivo*. As an intracellular RNA sensor, TLR8 triggers autoimmune inflammation when activated by autologous mitochondrial RNA (10). It also recognizes viral RNA and UR/URR motifs in bacterial RNA (11, 12). The balance of TLR8 signaling is critical to the maintenance of homeostasis, as the lack or impairment of TLR8 signaling is detrimental in some diseases such as multiple sclerosis (13). As a member of pattern recognition receptors, the role of TLR8 in TB is growing in interest to investigators. Some reports indicate a genetic association between *TLR8* and TB (14–16), and a functional role for TLR8 in the survival of *Mtb* has been published from a genome-wide siRNA screen (17). Previously, we found that inhibition of TLR8-mediated signaling promotes Bacillus Calmette–Guérin (BCG) induced apoptosis in human THP-1 cells (18). To further investigate the role of TLR8 in TB, we produced an h*TLR8* transgenic mouse with a macrophage-specific promoter as a study model. Furthermore, with a specific agonist of human TLR8, TL8-506, we tested the adjuvant potential of TLR8 agonist in this mouse model.

## Materials and Methods

### Ethics Statement

The animal studies were conducted under the approval of Institutional Animal Care and Use Committee of the Institute of Laboratory Animal Sciences, CAMS&PUMC, using the recommendations from the Guide for the Care and Use of Laboratory Animals of the institute (approval nos. ZLJ16001 and ZLJ16007).

### Animals

All mice were housed under SPF-level conditions. *TLR8* human cDNA ORF (NM_138636) (OriGene) with a macrophage-specific synthetic promoter SP146+ P47 (19, 20) was transferred into C57BL/6 mice to acquire the transgenic mice. *Ifnar1*^−/−^ mice (21) were crossed with *TLR8* transgenic mice. Then, the F1 generation was inbred to acquire *Ifnar1*^−/−^ *TLR8* mice.

For the immunogenicity study, 6-week-old female mice were vaccinated intramuscularly with PBS, OVA (Sigma)-aluminum hydroxide gel (Invivogen), or OVA-aluminum hydroxide gel-TL8-506 (Invivogen). At the indicated time points, mice were euthanized, splenocytes and serum were collected for antigen-specific IFNγ enzyme-linked immunospot (ELISPOT), flow cytometry, and antibody responses.

For the infection study, 8-week-old male and female mice (*TLR8* transgenic mice or C57BL/6 WT littermates) were intravenously challenged with 2 × 10^6^ CFU of *M. tuberculosis* H37Rv in 200 µL of PBS. The bacterial load of lung, liver, and spleen was determined 4 weeks following challenge by BACTEC MGIT 960 system and plating on solid LJ medium. Lung, liver, and spleen tissues were fixed in 4% formaldehyde for at least 4 days. Then, paraffin-embedded tissue sections were cut and H&E stain was performed.

For the protection study, 6-week-old female *TLR8* transgenic mice were vaccinated intramuscularly with PBS, ESAT6 (Abcam)-aluminum hydroxide gel, or ESAT6-aluminum hydroxide gel-TL8-506. The second immunization was performed 4 weeks later. Mice were challenged with 10^5^ CFU of *M. tuberculosis* H37Rv in 200 µL of PBS 4 weeks following the second vaccination. The bacterial load of lung, liver, and spleen was determined 10 weeks post challenge by fast detection through BACTEC MGIT 960 system and plating on solid LJ medium. Splenocytes and serum were collected for flow cytometry and cytokine examination (ProcartaPlex, eBioscience). In the infection and protection study, the experimental scheme was approved by institutional the ABSL-3 laboratory administration committee, and the infection procedure and housing of infected animals was conducted in the ABSL-3 laboratory of the Institute of Laboratory Animal Sciences, CAMS&PUMC.

### Bacterial Burden

Lung, liver, and spleen tissues were homogenized in 1 mL PBS. Serial dilutions of the tissue homogenate were added into MGIT 960 tubes to be cultured and monitored by the MGIT 960 system, or plated on LJ medium. The report by MGIT 960 system of time-to-detection (TTD) and Growth Unit was used to evaluate the bacilli load, and CFU was enumerated following incubation of the LJ plates at 37°C for 21–28 days.

### Cell Preparation and Flow Cytometry

Spleen tissue was gently mashed through a 70 µm nylon membrane filter in lymphocyte separation medium to obtain single cell suspensions. Lymphocytes were collected following centrifugation. Following cell count and dilution, lymphocytes were treated with anti-mouse CD16/CD32 antibodies (Mouse BD Fc Block, BD Biosciences) for 15 min and then stained with CD4-FITC (clone RM4-5, eBioscience), CD8-PerCP-Cy5.5 (clone 53-6.7, eBioscience), CD62L-PE (clone MEL-14, BD Biosciences), and CD44-APC (clone IM7, eBioscience) antibodies. The intracellular staining of TLR8 (clone 112H7.15, Dendritics) or BCL2 (clone BCL/10C4, Biolegend) was performed according to the instructions of the manufacturer (Fixation/Permeabilization Solution Kit, BD Biosciences). Following staining, cells were washed with PBS, fixed in 1% formaldehyde, and analyzed on BD Accuri C6 Plus or FACSAria (BD bioscience). Frequency of target cell types was calculated by FlowJo software.

### Detection of Antigen-Specific IFNγ Release by ELISPOT

Antigen-specific IFNγ-producing cells in the spleen were determined by the ELISPOT. Lymphocytes were separated from the spleen as described above and seeded at a concentration of 0.8 × 10^6^ per well on mouse IFNγ pre-coated PVDF plates (DAKEWE Biotech). Cells were then stimulated with 100 µg/mL OVA or 40 µg/mL ESAT-6 at 37°C for 16 h. Spot forming units were determined with ELISPOT plate reader (Cellular Technology).

### Antibody ELISA

Antibody levels in mouse serum were estimated with OVA (100 µg/mL) for coating and anti-mouse IgG1, IgG2a (Abcam) HRP-conjugated antibodies for detection.

## Results

### Expression of Human TLR8 in Mice Results in Higher Bacilli Load

**Figure 1:**
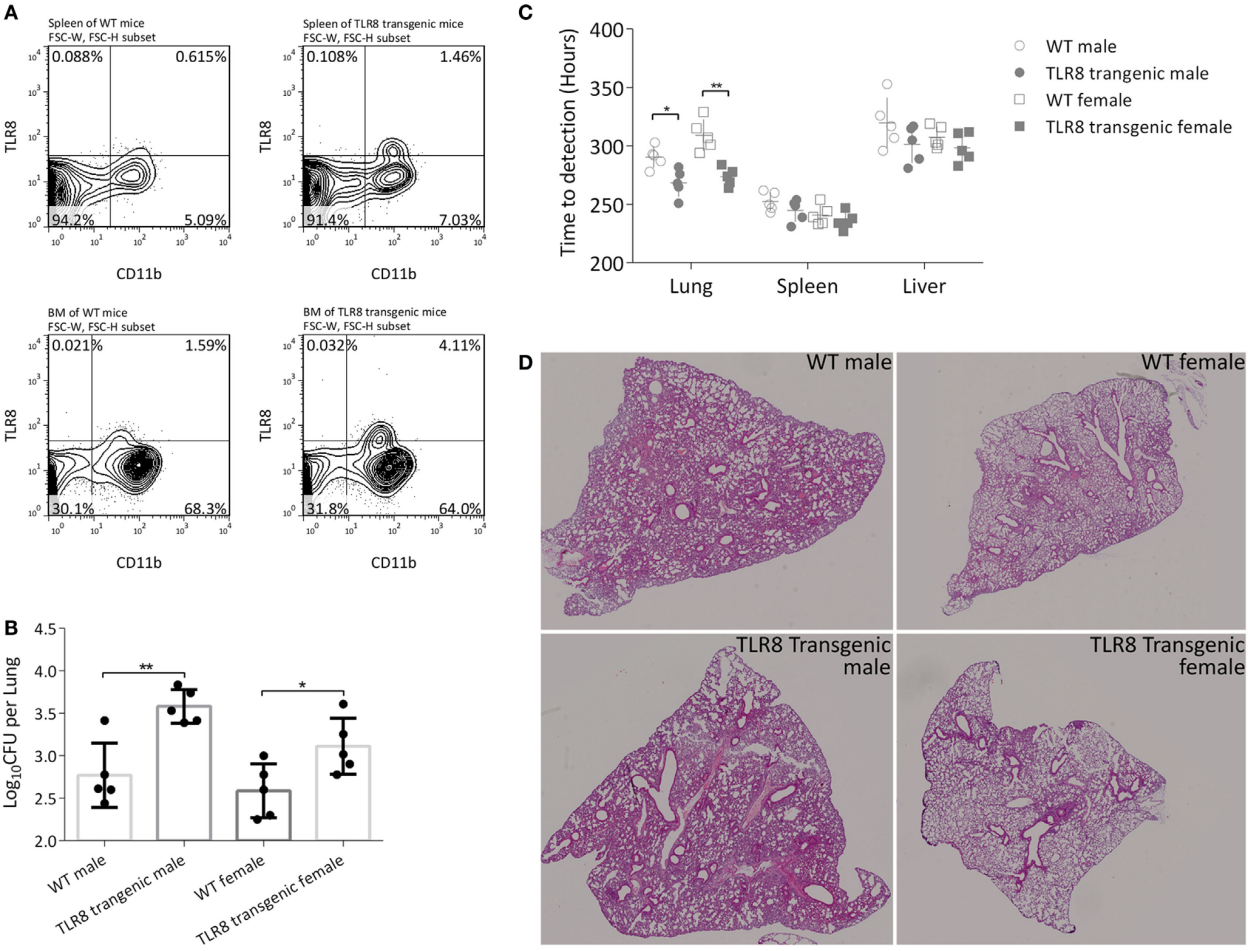
Expression of human TLR8 in C57BL/6 mice results in higher bacilli load in tissues. **(A)** Proportion of TLR8 positive monocytes (human TLR8^+^ CD11b^+^) in the spleen (top) and bone marrow (bottom) of C57BL/6 (WT, left) or human *TLR8* transgenic mice (right). **(B)** CFU of lungs 4 weeks post *Mtb* infection. **(C)** Time-to-detection (TTD) by the MGIT 960 system in lung, spleen, and liver 4 weeks post *Mtb* infection. CFU are log_10_-transformed and TTD are transformed into hours, prior to a Student’s *t*-test or one-way ANOVA with a Bonferroni posttest. Data are representative of two independent experiments, each with five mice per group. **(D)** Lung sections of WT mice or *TLR8* transgenic mice were stained with H&E 4 weeks post *Mycobacterium tuberculosis (Mtb)* infection. *P < 0.05, **P < 0.01.

To acquire a suitable mouse model to study human TLR8 function *in vivo*, we transferred human *TLR8* ORF with a macrophage-specific synthetic promoter SP146+ P47 (19, 20) into C57BL/6. This transgenic mouse expresses human TLR8 with similar distribution as in humans, but at a relative low level to avoid spontaneous disorders. TLR8 protein expression was measured and quantified by flow cytometry in several main organs, including the spleen and bone marrow (Figure 1A). Furthermore, both sexes were monitored for more than 10 months, and no signs of disorders, such as inflammation in joints or corneal defects, were observed (7).

We then challenged 8-week-old male and female mice (*TLR8* transgenic mice or WT littermates) with *Mtb* H37Rv and examined disease progression after 4 weeks. Bacilli load was quantified by calculating CFU in lungs. Serial dilutions of tissue homogenate were plated on Lowenstein-Jensen medium slants and cultured for 4 weeks. There was a significantly higher bacteria load in the lungs of *TLR8* transgenic mice compared to C57BL/6 mice, for both male and female (Figure 1B). We also evaluated bacterial load using the BACTEC MGIT 960 system. With accurate detection of oxygen consumption, the system generates two readouts for each sample, TTD and Growth Unit, much faster than by using solid medium like L-J slant or 7H10 agar. In the lung, spleen, and liver, positive for samples were detected faster from *TLR8* transgenic mice than from C57BL/6 mice, which corroborates our CFU results (Figure 1C). Considering the high throughput, rapid culture, and high automation level, we propose that the readouts of the MGIT 960 system can be used to evaluate the bacilli load in tissues in experimental applications.

Although the bacilli load in lungs is quite different between *TLR8* transgenic mice and WT mice, the pathological lesions are similar, which suggests that expression of human TLR8 in C57BL/6 mice may permit higher *Mtb* load in tissues (Figure 1D).

Male mice in both groups were more susceptible to *Mtb* and developed more severe disease (Figures 1B,D). This TB gender difference in C57BL/6 mice we observed was similar to that of human adults in population. The regulatory activities of steroid sex hormones such as estrogens and testosterone on immune cells are argued to partially explain the phenomenon (22, 23). *TLR8* polymorphisms are also reported to be associated with TB gender susceptibility difference (16). However, in this study, expression of human TLR8 did not alter the TB gender difference in C57BL/6 mice, either on bacilli load or on pathological lesions.

### Vaccine Formulated with ESAT-6, Aluminum Hydroxide, and TLR8 Agonist Provides Better Protection against *Mtb* Challenge

**Figure 2:**
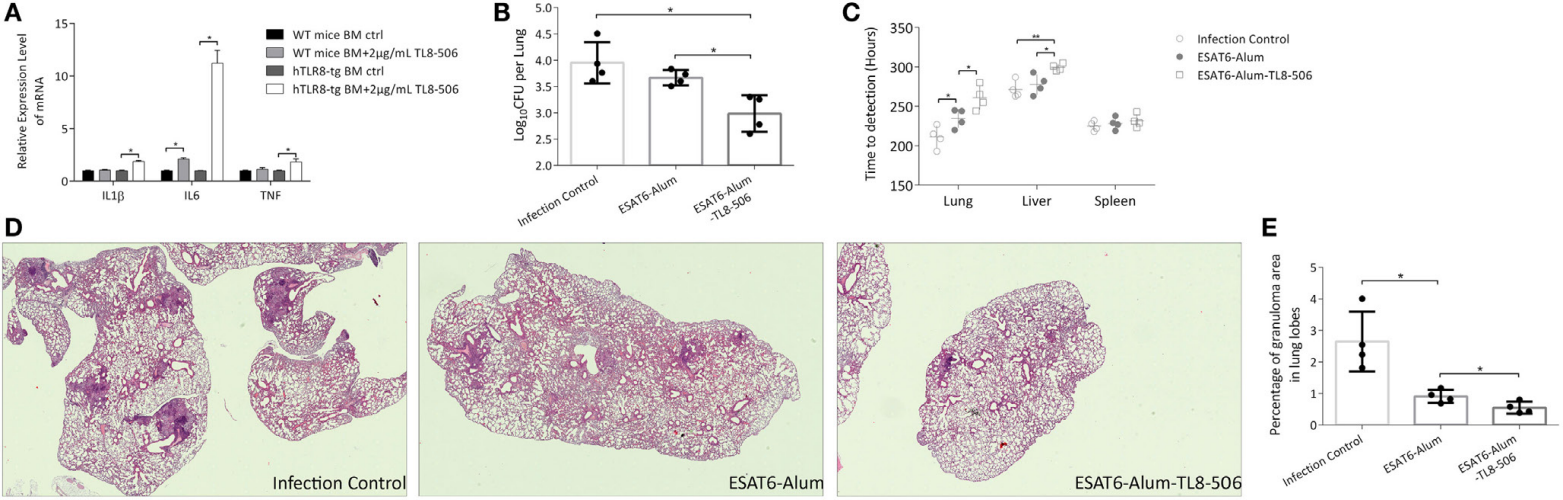
*TLR8* transgenic mice immunized with TLR8 agonist carry lower bacilli load and display ameliorated pathological lesions in tissues following a challenge. **(A)** Bone marrow cells were cultured and treated *ex vivo* as indicated for 12 h before the mRNA level of IL1β, IL6, and TNF was determined. Three mice per group. *P < 0.05, by Student’s *t*-test. **(B)** CFU (log_10_-transformed) of lungs in differently immunized *TLR8* transgenic mice 10 weeks post *Mycobacterium tuberculosis* challenge. **(C)** Time-to-detection (transformed into hours) by the MGIT 960 system in lung, spleen, and liver. **(D)** Lung sections stained with H&E. **(E)** Percentage of granuloma area in lung lobes determined by NanoZoomer S60 (Hamamatsu) and software, 3–5 sections per mice. *P < 0.05, **P < 0.01, by one-way ANOVA with a Bonferroni posttest. Data are representative of two independent experiments, each with four mice per group.

To further study human TLR8 function in our mouse model, we designed a TB vaccine formula and measured its immune effect and protection level *in vivo*. This formula consists of *Mtb* antigen ESAT6, aluminum hydroxide gel (alum), and a human TLR8 specific ligand, TL8-506. TL8-506 is a benzoazepine compound, an analog of the TLR8 agonist VTX-2337, but has a much lower EC50 (30 nM) (24, 25). The specificity of TL8-506 is higher than the imidazoquinoline R848 and the thiazoquinolone CL075 (26). Bone marrow cells of *TLR8* transgenic mice, but not WT mice, readily respond to the *ex vivo* stimulation with TL8-506 (Figure 2A). As an approved and widely used adjuvant, alum induces a Th2 response. We used alum to prolong the stimulation of the injected immunogen by the “depot” effect of alum, and to adsorb both ESAT6 and TL8-506 during formulation (27). To determine the adsorption capacity of alum for TL8-506, the supernatant of the mixture at different ratios following centrifugation was assayed in THP-1 cells to detect the activity of unbound agonist (Figure S1 in Supplementary Material). The adsorption capacity of TL8-506 was more than 0.1 mg per mg of alum.

Four weeks following a prime-boost immunization regimen of ESAT6-Alum, ESAT6-Alum-TL8-506, or sham injection, *TLR8* transgenic mice were challenged with *Mtb* H37Rv intravenously. Ten weeks post infection, the disease progression in each group was examined. The combination of ESAT6-Alum-TL8-506 provides better protection against *Mtb* challenge compared to ESAT6-Alum. The CFU in the lungs of mice treated with ESAT6-Alum-TL8-506 was significantly lower than that in the ESAT6-Alum group (Figure 2B). Readouts of the MGIT 960 system, TTD and Growth Unit, reveal significantly lower bacterial load in the lungs and livers of mice treated with ESAT6-Alum-TL8-506 compared to mice treated with ESAT6-Alum alone. No significant difference in any group was measured in the spleens (Figure 2C; Figure S2 in Supplementary Material).

In lung sections of infected control mice stained with hematoxylin-eosin, coalescing foci of tuberculous pneumonia, interstitial granulomatous infiltrates, alveolitis with thickened alveolar walls, and multiple compact granulomas surrounded by foamy macrophages are visible (Figure 2D). Vaccination with ESAT6-Alum-TL8-506 significantly ameliorated the tuberculous pneumonia and alveolitis and reduced the numbers and the area of granulomas, compared with the ESAT6-Alum group. To illustrate the histopathological change on granulomas in each group, we calculated the area of each granuloma on different lobe sections, and divided the sum of granuloma area by the section area to obtain the percentage of granuloma area in lung lobes for each mouse. Mice in the ESAT6-Alum-TL8-506 group had a significantly lower percentage of granuloma area than mice in the other two groups (Figure 2E). At another time point, 16 weeks post infection, the CFU (Figure S3A in Supplementary Material) and granuloma area (Figures S3B,C in Supplementary Material) in the lungs of ESAT6-Alum-TL8-506 group remained lower than the other groups, although the difference between groups was much smaller than at the early time point.

**Figure 3:**
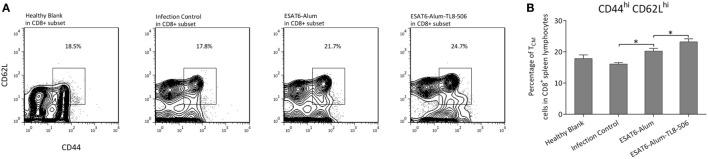
*TLR8* transgenic mice immunized with TLR8 agonist display a higher proportion of central memory CD8^+^ T cells following a challenge. **(A)** Representative flow cytometry and **(B)** proportions of CD44^hi^CD62L^hi^ subset in CD8^+^ splenic lymphocytes of differently immunized *TLR8* transgenic mice 10 weeks post *Mycobacterium tuberculosis* challenge. *P < 0.05, by one-way ANOVA with a Bonferroni posttest.

We characterized immune cell composition in each group and demonstrated a significant difference in the proportion of memory T cells among groups. Central memory T cells, which have a high proliferative capacity, are considered an important source of acquired resistance. One primary flaw of BCG is its inability to effectively establish central memory T cells (28). Here, central memory CD8^+^ T cells (T_CM_) expressing CD44^hi^CD62L^hi^ were significantly increased in the ESAT6-Alum-TL8-506 group (Figures 3A,B). The changes in the proportion of memory T cells can be attributed to the direct activation by ESAT6-Alum-TL8-506, or simply reflect a consequent effect of milder illness in those mice. To tease this out, we examined the change in memory T cell subsets in vaccinated mice without an *Mtb* challenge.

### Antigen Ovalbumin with TLR8 Agonist Induces a Long Lasting Immune Memory

**Figure 4:**
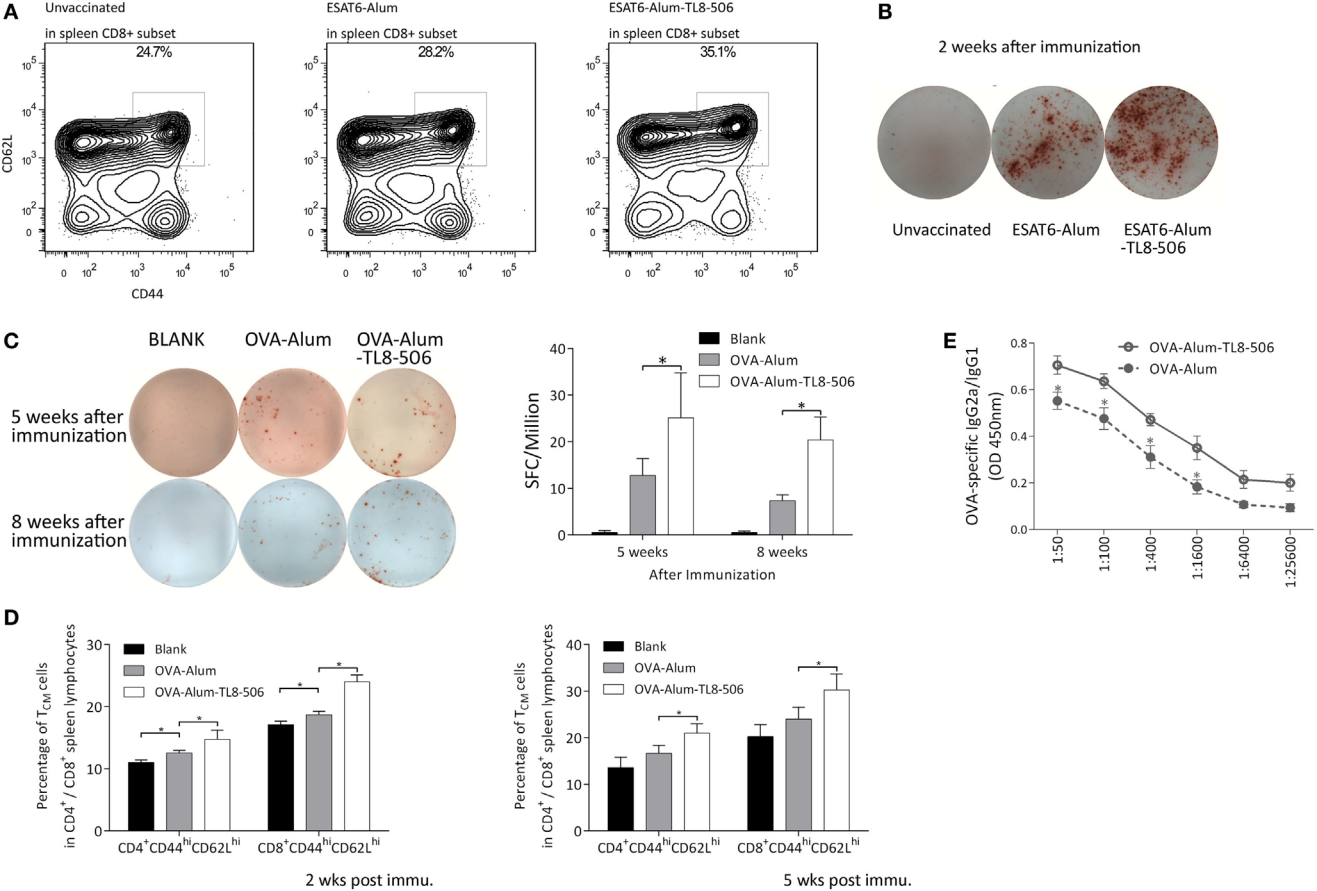
Formulation with TLR8 agonist induces a long-lasting immune memory. **(A)** Representative flow cytometry of CD44^hi^CD62L^hi^ subset in CD8^+^ splenic lymphocytes of *TLR8* transgenic mice 2 weeks post differential immunization with ESAT6. **(B)** Representative image of ESAT6-specific IFNγ-secreting splenic cells in *TLR8* transgenic mice 2 weeks post differential immunization with ESAT6. **(C)** OVA-specific IFNγ-secreting splenic cells in differently immunized *TLR8* transgenic mice 5 or 8 weeks post immunization determined by ELISPOT. Five mice per group. *P < 0.05, by one-way ANOVA with a Bonferroni posttest. **(D)** Proportions of CD44^hi^CD62L^hi^ subset in CD4^+^ or CD8^+^ splenic lymphocytes of *TLR8* transgenic mice 2 or 5 weeks post differential immunization with ova. **(E)** The ratio of OVA-specific IgG2a to IgG1 in sera of differently immunized *TLR8* transgenic mice 2 weeks following immunization. *P < 0.05, by Student’s *t*-test.

Two weeks post immunization with ESAT6-Alum or ESAT6-Alum-TL8-506, the immune response induced was characterized. The proportion of CD8^+^ T_CM_ in the ESAT6-Alum-TL8-506 group was higher than in the other groups (Figure 4A). Furthermore, the population of ESAT6-specific IFNγ-secreting cells in the spleen of the ESAT6-Alum-TL8-506 group was larger than in other groups (Figure 4B).

One important mechanism in the establishment of T_CM_ is the participation of antiapoptosis molecules like BCL-2 in the maintenance of T_CM_. Therefore, we analyzed the expression of BCL-2 in the spleen CD8^+^ T cells (29, 30). However, no significant differences in the expression level of BCL-2 between ESAT6-Alum and ESAT6-Alum-TL8-506 groups were observed (Figure S4 in Supplementary Material).

To further characterize the adjuvant effect of the TLR8 agonist, we used ovalbumin as a model immunogen, combined with Alum or Alum-TL8-506, to immunize the *TLR8* transgenic mice. Then, we analyzed the percentage of IFNγ secreting cells in the spleen of each group by ELISPOT. Five and eight weeks post inoculation, the IFNγ-secreting cells in the spleen of the OVA-Alum group decreased dramatically compared to the OVA-Alum-TL8-506 group, suggesting that TL8-506 helps establish a persistent CD4^+^ T cell response (Figure 4C).

Next, we examined the proportion of memory T cells in each group at different time points (Figure 4D). Two and five weeks post inoculation, flow cytometric analysis reveals a higher percentage of CD44^hi^CD62L^hi^ T_CM_ in the OVA-Alum-TL8-506 group compared to the OVA-Alum group. This is consistent with the results of ESAT-6 vaccination and challenge experiments above. The establishment of a larger CD44^hi^CD62L^hi^ T_CM_ population is an important effect resulting from TLR8 agonist addition to the vaccine.

As alum mainly induces a Th2 response, we examined whether the addition of TL8-506 enhanced a Th1 humoral response skewed toward IgG2a class-switching, which is protective against intracellular infections (31). Two weeks post inoculation, serum from each group was serially diluted to assess levels of OVA-specific IgG1 and IgG2a by ELISA. Inoculation of OVA-Alum-TL8-506 increased the ratio of IgG2a/IgG1, indicating TL8-506 promotes this isotype switching (Figure 4E).

### Type I IFN Receptor Signaling Is Critical in the Establishment of T_CM_ Promoted by TLR8 Agonist

**Figure 5:**
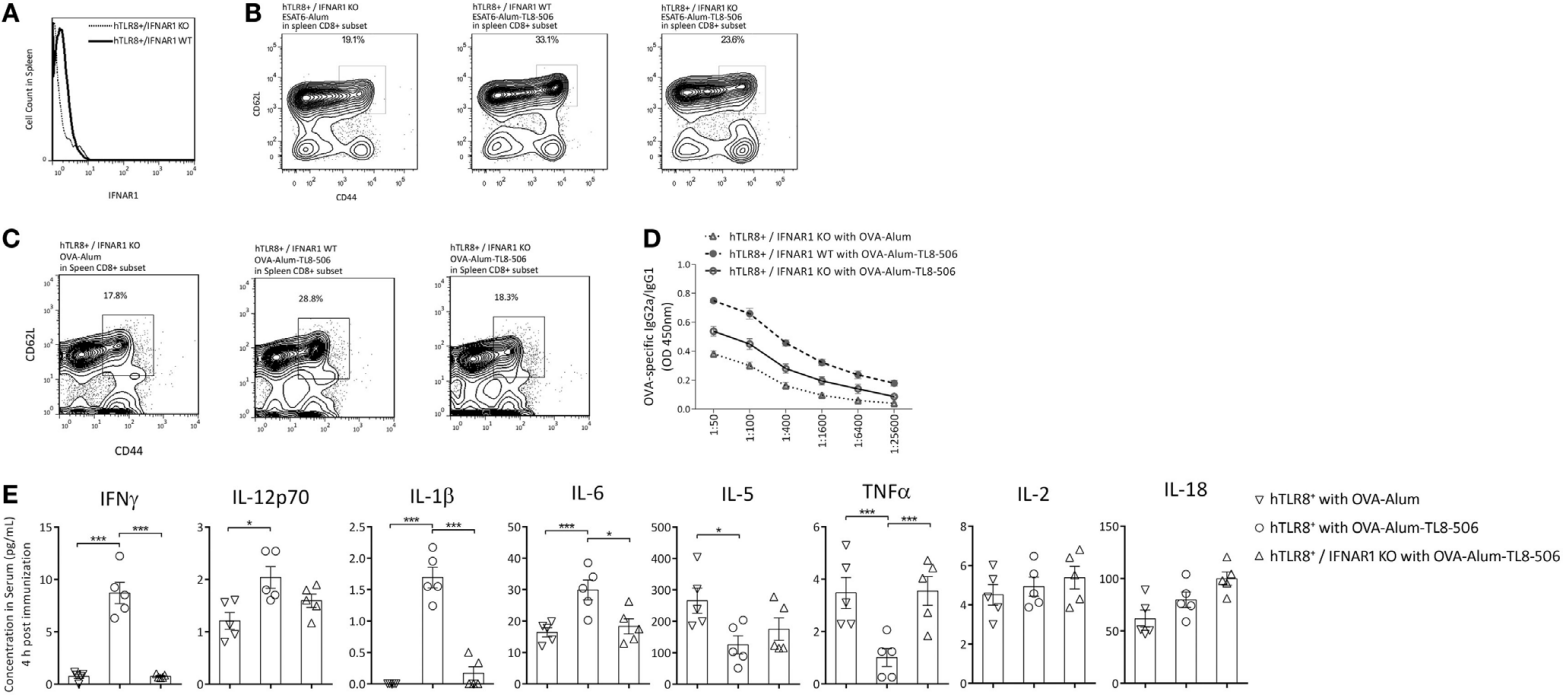
Type I IFN receptor signaling is critical in the establishment of T_CM_ promoted by TLR8 agonist. **(A)** Histograms of PE-IFNAR1 antibody staining in spleen of hTLR8^+^/IFNAR1 KO mice and hTLR8^+^/IFNAR1 WT mice. **(B)** Proportions of CD44^hi^CD62L^hi^ subset in CD8^+^ splenic lymphocytes of differently immunized hTLR8^+^/IFNAR1 KO mice (left and right) and hTLR8^+^/IFNAR1 WT mice (middle) 2 weeks following immunization with ESAT6 or **(C)** with OVA. **(D)** The ratio of OVA-specific IgG2a to IgG1 in sera of hTLR8^+^/IFNAR1 KO mice and hTLR8^+^/IFNAR1 WT mice 2 weeks following immunization. Three mice per group. **(E)** Cytokine levels in sera 4 h post differential immunization with ova, determined by ProcartaPlex (eBioscience). *P < 0.05, **P < 0.01, ***P < 0.001, by one-way ANOVA.

TLR8 is known to signal through MyD88 and then IRAK-4 or IRF-7, activate the NFκB, MAPK, or type I IFN signaling pathways. Type I IFN pathway plays an important role in the innate response induced by a TLR4 agonist, GLA-SE, mainly on IFNγ production, Th1 induction, and Th2 counter-regulation (2). Therefore, we explored the role of IFNAR1, the receptor of type I IFN, in the immune response enhanced by TLR8 agonist. IFNAR1 KO mice (C57BL/6 background) were mated with our *TLR8* transgenic mouse. Then, the F1 generation was inbred to acquire *Ifnar1*^−/−^*TLR8* mice (hTLR8^+^/IFNAR1 KO) (Figure 5A). The hTLR8^+^/IFNAR1 KO mice and hTLR8^+^/IFNAR1 WT mice were vaccinated with ESAT6-Alum or ESAT6-Alum-TL8-506 (Figure 5B), OVA-Alum or OVA-Alum-TL8-506 (Figure 5C). The resulting immune responses were characterized 2 weeks later.

The percentage of CD44^hi^CD62L^hi^ T_CM_ in the spleen was significantly lower in the hTLR8^+^/IFNAR1 KO group, compared with the hTLR8^+^/IFNAR1 WT group, indicating an important role of type I IFN signaling in the establishment of T_CM_ promoted by TL8-506 (Figures 5B,C).

OVA-specific IgG1 and IgG2a levels in sera were also determined by ELISA in these groups. The Th1 humoral response enhanced by TL8-506 was impaired due to the lack of IFNAR1 in hTLR8^+^/IFNAR1 KO mice (Figure 5D). Therefore, the role of type I IFN signaling in this model is similar to that of the TLR4 agonist GLA-SE elicited response (2). The Th1 induction and Th2 counter-regulation by GLA-SE is also partially dependent on type I IFN production.

To get acute profile of immune response, we examined cytokines in the serum 4 h post immunization with antigen ova in hTLR8^+^/IFNAR1 KO mice and hTLR8^+^/IFNAR1 WT mice. The levels of IFNγ, IL-12p70, IL-1β, and IL6 were significantly increased with the addition of TLR8 agonist, while IL-5 and TNF were suppressed. The phenotype caused by TL8-506 was reversed in hTLR8^+^/IFNAR1 KO mice, indicating a key role for type I IFN receptor signaling (Figure 5E).

## Discussion

TLR8 agonists have promising potential applications in different diseases. Recently, using humanized *TLR8* mice, Dowling and colleagues reported that TLR8 agonist polymersomes induced high IL-12p70 levels and newborn DC maturation profiles similar to those induced by BCG (26). Our work further validates the protection improvement by TLR8 agonist against *Mtb* challenge, and more specifically reveals the role of TLR8 activation in the establishment of T_CM_ post vaccination.

Several reports indicate an association between TLR8 and TB; however, the role of TLR8 in the process and pathogenesis of TB is still not well understood. One important obstacle is the lack of relevant animal models for *in vivo* research. The results from *ex vivo* or *in vitro* experiments cannot fully reveal the role of TLR8 in this complex context. Using our human *TLR8* transgenic mouse, we demonstrate the effect of TLR8 on TB disease progression for the first time. Different terms are often used by researchers to describe responses of different hosts to *Mtb* infection: susceptible versus resistant (such as the A/J versus C57BL/6J mouse strain) (32), and permissive versus sensitive (such as mice versus guinea pig). Here, we consider mice carrying human TLR8 as more permissive than wild type C57BL/6 mice, as the former has a higher bacilli load, but similar pathological lesions compared to the latter. Furthermore, by utilizing next generation sequencing on different tissues and cell types, we have been seeking the mechanisms underlying the role of TLR8 in disease progression.

Type I IFN signaling pathway is an important downstream branch of TLR8 signaling. Aside from its role in host antiviral immunity, other characteristics, including responses to bacterial infection, are usually detrimental to protective immunity (33, 34). In the case of TB, impaired STAT1-mediated type I IFN signaling in DCs leads to increased production of IL-12 and increased differentiation of T cells toward Th1 (35). IFNAR1-deficient mice largely control *M. tuberculosis* more efficiently than WT animals (36, 37). However, in the absence of IFNγ signaling, type I IFN suppresses the permissive alternatively activated phenotype of macrophages and contributes to host protection against *Mtb* (38). Therefore, the higher bacteria load in lungs of *TLR8* transgenic mice may be partially attributed to the enhancement of downstream type I IFN signaling.

On the other hand, type I IFN links innate and adaptive immunity. It inhibits the death of activated CD4 T cells, and their role in priming adaptive T cells has been demonstrated in different vaccination schemes (39). Recombinant type I IFN exhibits direct adjuvant activity *in vivo*, enhancing humoral and T cell responses (40, 41). Some TLR and non-TLR agonists require the activity of type I IFN to exert their adjuvant effect (42). But other adjuvants, such as MF59 (TLR independent) and Pam3CSK (TLR2), do not induce type I IFN for their adjuvanticity (43). The results of antigen ESAT6 and ovalbumin demonstrate that type I IFN is critical for the adjuvanticity of TL8-506. Indeed, IFNAR1 knockout caused a marked reduction in both the frequency of CD44^hi^CD62L^hi^ T_CM_ and in Th1 skewing measured by IgG2a/IgG1 ratio. However, the role of type I IFN downstream of TLR8 activation in the protective efficacy of the ESAT-6 formulation needs further investigation in hTLR8^+^/IFNAR1 KO mice with *Mtb*.

The BACTEC MGIT 960 system has been considered a powerful diagnostic tool in early detection and drug resistance screens clinically. As a rapid culture system based on liquid medium, it delivers quantitative readouts directly associated with the growth of *Mtb*. The fluorometric signal detection provides low detection threshold (<10 bacilli), and the high level of automation provides high reproducibility (44). The performance of discriminative ability and detection threshold is more powerful than Xpert MTB/RIF, a clinical quantitative PCR system (44). Therefore, TTD of *Mtb* in BACTEC systems was hypothesized as a viable alternative to colony counting (45). In our study, we not only confirmed the performance of TTD in discrimination of different bacilli load but also validated the correlation between Growth Unit and bacilli load in samples. Considering the high performance and ease of use, the BACTEC MGIT 960 system could facilitate TB research in medical institutions.

Early post vaccination, TLR8 agonist induces higher proportions of T_CM_ in the spleen. Memory T cells are major subsets of immune cells considered to contribute to vaccine efficacy and longevity of protection. Two populations of memory T cells, T_CM_ and T_EM_, differ in properties, including distribution, surface markers, cytokine secretion, and speed of response (46, 47). T_CM_ responses are faster than T_EM_, but several studies suggest that in TB, T_EM_ accounts for a higher proportion of the acquired resistance (48, 49). However, when looking at the spleen instead of lung or blood, the percentage of T_CM_ is significantly greater, suggesting the existence of a potential reservoir in the spleen (48). Although the roles of memory T cells as vaccine targets are still controversial, T_CM_ are still considered as an important marker of acquired immunity and vaccine efficacy. In our study, the formation of T_CM_ in the spleen demonstrated an important aspect of the immune responses engaged by the TLR8 agonist.

In this study, we demonstrated the beneficial effect of human TLR8 activation on *Mtb* antigen induced protective immunity. Seeking combinations of different TLR agonists for better adjuvanticity in this animal model could be an important next step of our research.

## Ethics Statement

The animal studies were conducted under the approval of Institutional Animal Care and Use Committee of Institute of Laboratory Animal Sciences, CAMS&PUMC, using the recommendations from the Guide for the Care and Use of Laboratory Animals of the institute (approval nos. ZLJ16001 and ZLJ16007).

## Author Contributions

LZ and CQ conceived and supervised the experiments; JT and MS performed the experiments; MS conducted the mice experiment; YX and YH conducted the pathology observations; GS and XL performed the flow cytometry; WD conducted the gene modifications of mice; JT, LZ, and CQ analyzed the data and drafted the manuscript. All authors reviewed and approved the manuscript.

## Conflict of Interest Statement

The authors declare that the research was conducted in the absence of any commercial or financial relationships that could be construed as a potential conflict of interest.

## Supplementary Material

Figure S1 Adsorption of TLR8 agonist TL8-506 to aluminum hydroxide. TL8-506 and Alum or PBS were thoroughly mixed by pipette for 5 min at different ratios, allowed to sit for 1 h, and then centrifuged at 14,000 × *g* for 5 min. The supernatant was added into the culture of THP-1 cells to detect the activity of unbound TLR8 agonist. The mRNA levels of TNF and IFNA was determined 12 h following treatment. N.S., no significance by Student’s *t*-test.

Figure S2 Growth Unit by the MGIT 960 system to evaluate bacilli load in tissues. Growth Unit (recorded at day 15 or 35 of culture in the system) by the MGIT 960 system in lung, spleen, and liver of differently immunized *TLR8* transgenic mice 10 weeks post *Mycobacterium tuberculosis* challenge. **P* < 0.05, ***P* < 0.01, by one-way ANOVA with a Bonferroni posttest.

Figure S3 *TLR8* transgenic mice immunized with TLR8 agonist had lower bacilli load and ameliorated pathological lesions in the lung following challenge. **(A)** CFU (log_10_-transformed) of lungs in differently immunized *TLR8* transgenic mice 16 weeks post *Mycobacterium tuberculosis* challenge. **(B)** Percentage of granuloma area in lung lobes determined by NanoZoomer S60 (Hamamatsu) and software, 3–5 sections per mice. **P* < 0.05, ***P* < 0.01, by one-way ANOVA with a Bonferroni posttest. Four mice per group. **(C)** Lung sections stained with H&E.

Figure S4 *TLR8* transgenic mice immunized by ESAT6-Alum and ESAT6-Alum-TL8-506 expressed similar levels of BCL-2 in spleen CD8^+^ T cells. Two weeks post immunization, single cell suspension of splenocytes was stained with CD8 and BCL-2 antibody following fixation and permeabilization. The levels of BCL-2 were determined by flow cytometry.
