# Supplementary figures and images for: Toll-Like Receptor 8 Agonist Strengthens the Protective Efficacy of ESAT-6 Immunization to Mycobacterium tuberculosis Infection
Source: Front Immunol. 2018 Jan 24;8:1972. doi: 10.3389/fimmu.2017.01972 (PMC5787779; doi:10.3389/fimmu.2017.01972)

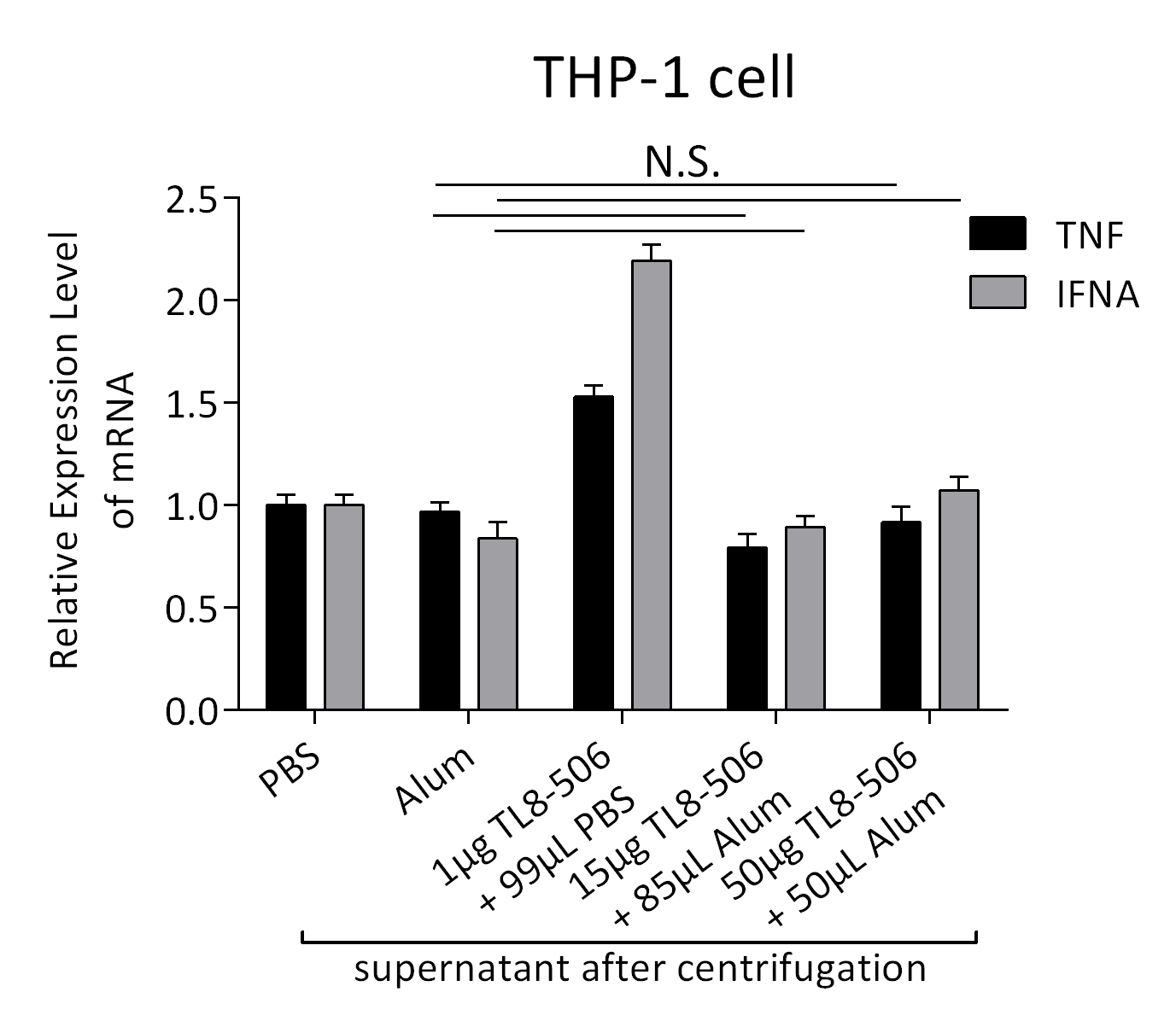

Supplement: Figure S1 — Adsorption of TLR8 agonist TL8-506 to aluminum hydroxide. TL8-506 and Alum or PBS were thoroughly mixed by pipette for 5 min at different ratios, allowed to sit for 1 h, and then centrifuged at 14,000 × g for 5 min. The supernatant was added into the culture of THP-1 cells to detect the activity of unbound TLR8 agonist. The mRNA levels of TNF and IFNA was determined 12 h following treatment. N.S., no significance by Student’s t-test. [file image_1.tif]

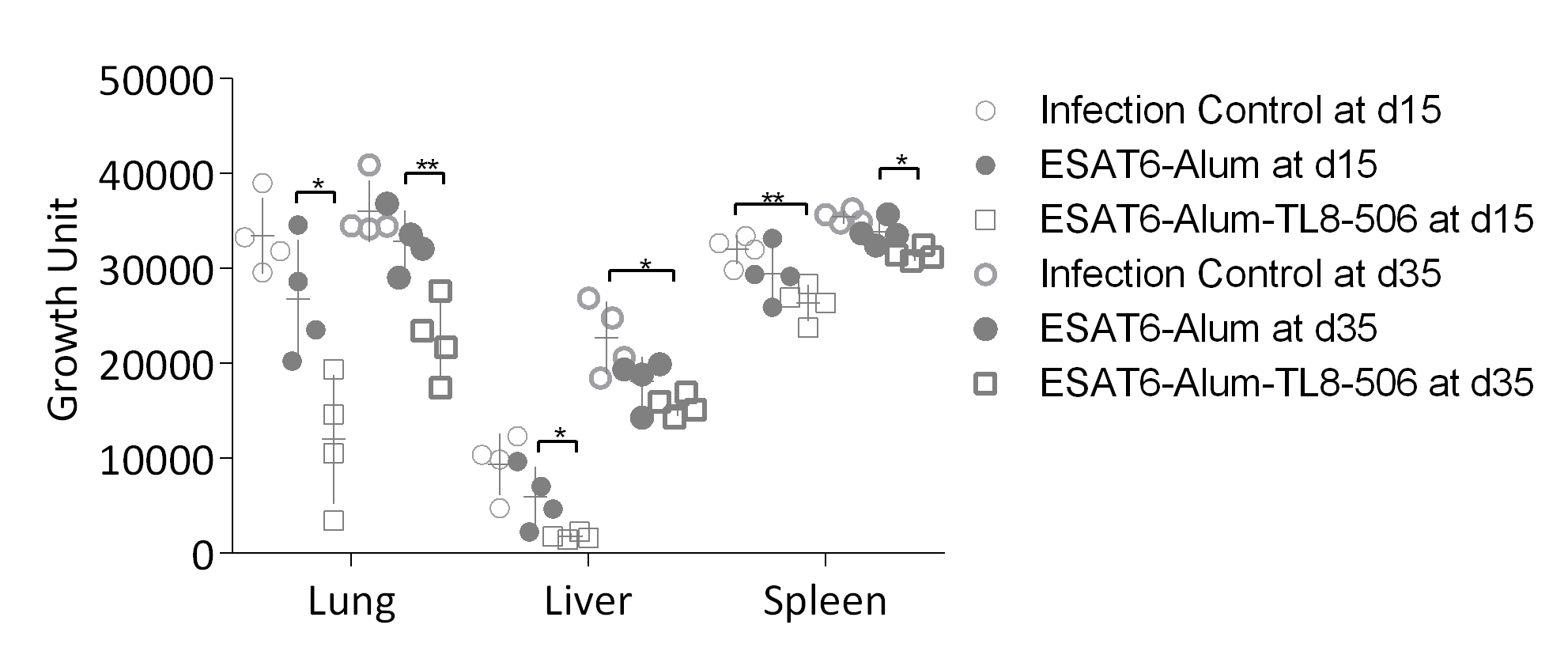

Supplement: Figure S2 — Growth Unit by the MGIT 960 system to evaluate bacilli load in tissues. Growth Unit (recorded at day 15 or 35 of culture in the system) by the MGIT 960 system in lung, spleen, and liver of differently immunized TLR8 transgenic mice 10 weeks post Mycobacterium tuberculosis challenge. *P < 0.05, **P < 0.01, by one-way ANOVA with a Bonferroni posttest. [file image_2.tif]

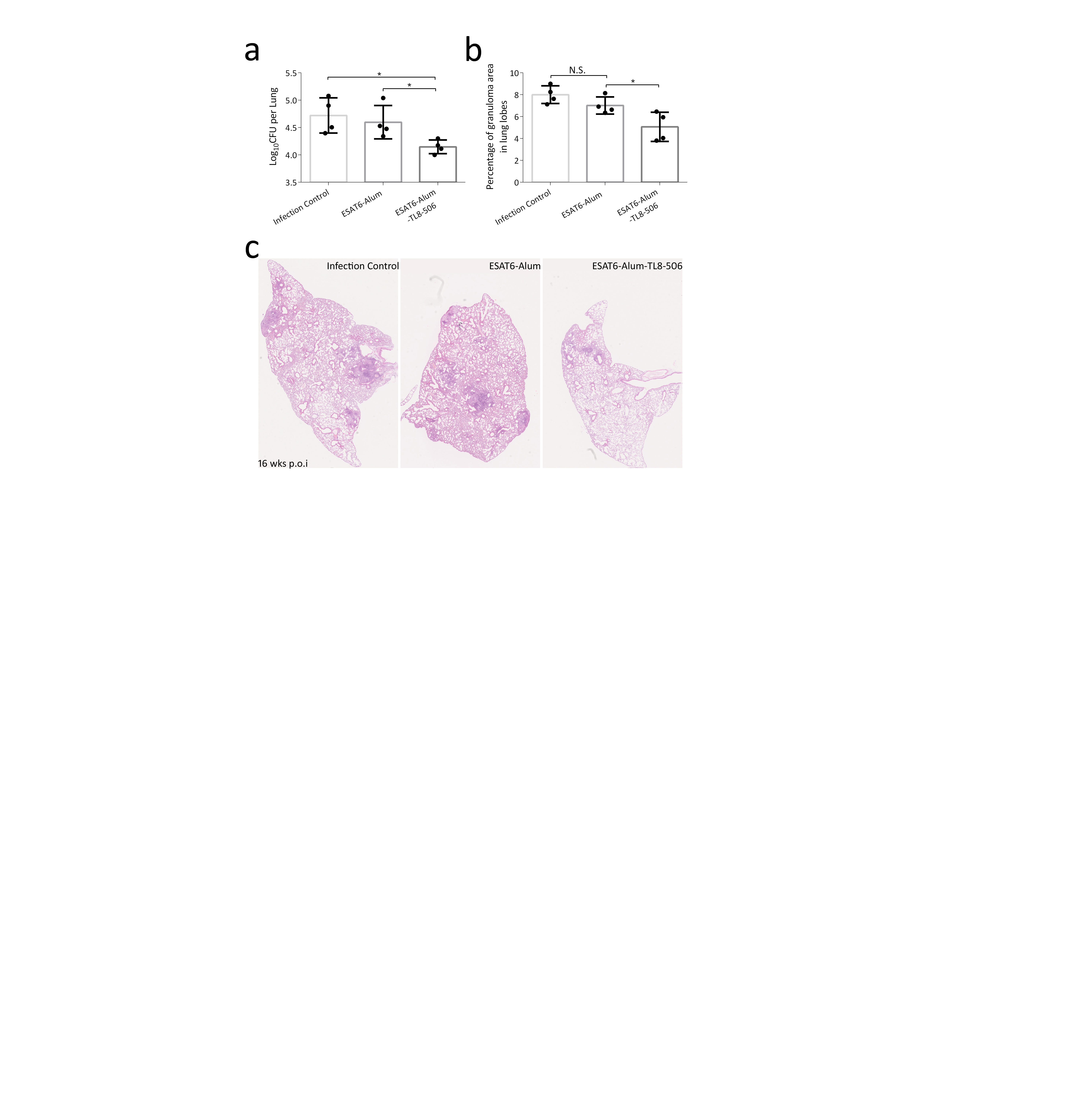

Supplement: Figure S3 — TLR8 transgenic mice immunized with TLR8 agonist had lower bacilli load and ameliorated pathological lesions in the lung following challenge. (A) CFU (log10-transformed) of lungs in differently immunized TLR8 transgenic mice 16 weeks post Mycobacterium tuberculosis challenge. (B) Percentage of granuloma area in lung lobes determined by NanoZoomer S60 (Hamamatsu) and software, 3–5 sections per mice. *P < 0.05, **P < 0.01, by one-way ANOVA with a Bonferroni posttest. Four mice per group. (C) Lung sections stained with H&E. [file image_3.tif]

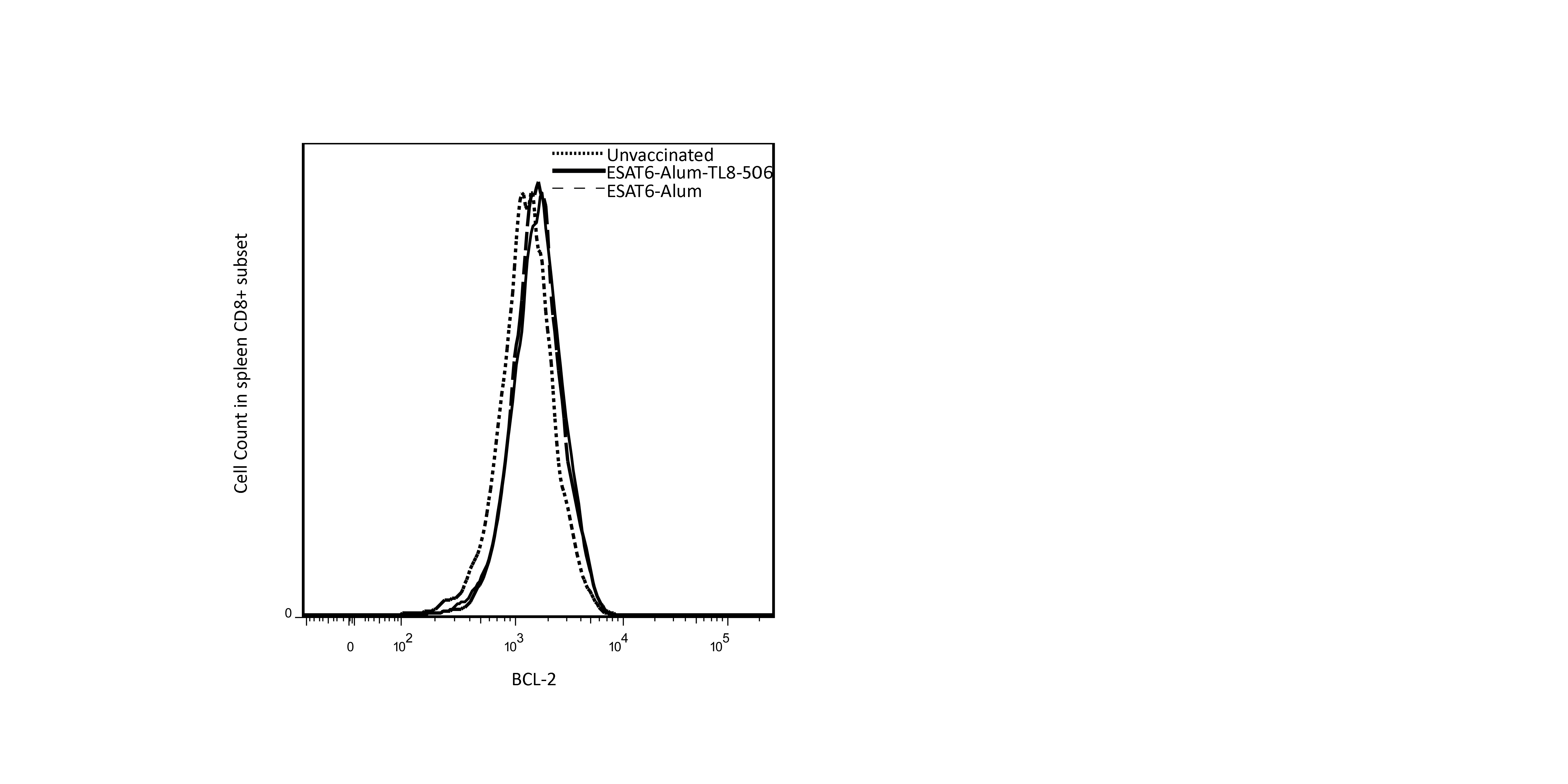

Supplement: Figure S4 — TLR8 transgenic mice immunized by ESAT6-Alum and ESAT6-Alum-TL8-506 expressed similar levels of BCL-2 in spleen CD8+ T cells. Two weeks post immunization, single cell suspension of splenocytes was stained with CD8 and BCL-2 antibody following fixation and permeabilization. The levels of BCL-2 were determined by flow cytometry. [file image_4.tif]
